# Effects of tail fat on recovery times of anesthesia with isoflurane in fat-tailed Iranian Lori-Bakhtiyari lambs

**Published:** 2015-09-15

**Authors:** Siavash Sharifi, Abbas Raisi Sarteshnizi, Farangis Sharifi, Elham Yousefian

**Affiliations:** 1 *Department of Clinical Sciences, School of Veterinary Medicine, Shahrekord University, Shahrekord, Iran; *; 2 *Department of Clinical Sciences, Faculty of Veterinary Medicine, Lorestan University, Khorramabad, Iran; *; 3 *Department ofMedical Sciences, Faculty of Nursing and Midwifery, Azad University, Kazeron Branch, Kazeron, Iran; *; 4 *Mazandaran Provincial Veterinary Service, Sari, Iran.*

**Keywords:** Anesthesia, Fat-tail, Isoflurane, Lori-Bakhtiyari lamb, Recovery times

## Abstract

In the present study, the effect of tail fat on recovery times in intact sheep and sheep with a ligated median sacral artery following similar anesthetic exposure with isoflurane was investigated. This study was performed using seven healthy fat-tailed Iranian Lori-Bakhtiyari ewe lambs. The lambs were anesthetized twice at two week intervals (the experiment was performed in two stages). After mask induction with isoflurane in 100% oxygen, sheep were intubated and anesthesia was maintained for 4 hr using a rebreathing system. Induction and extubation times and time to sternal recumbency and attempts to stand were recorded during anesthetic induction and recovery (Stage 1). Two weeks later, prior to the second anesthesia, the median sacral artery (MSA) was ligated under epidural anesthesia in sheep. All sheep were anesthetized as mentioned above (Stage 2). No significant differences were observed for the induction time between two stages (*p* > 0.05) but extubation, sternal recumbency and attempts to stand times were significantly longer in intact sheep (Stage 1) after 4 hr anesthesia with isoflurane (*p *< 0.05). Recovery time was decreased following MSA ligation in fat-tailed sheep, which suggested that body fat had a major role in the recovery time of isoflurane in sheep. We developed an animal model to investigate fat drug solubility of isoflurane gas. Therefore, using less-soluble in fat anesthetics is better than high-soluble anesthetics for prolonged anesthesia to decrease postoperative complication in obese patient.

## Introduction

Obesity is a growing challenge for anesthesiologists because of many problems including an increased incidence of intra- and postoperative atelectasis,^1^ difficulty in tracheal intubation,^2^ an increase in airway resistance that may resemble asthma,^3^ an increased capacity to metabolize anesthetics such as halothane or enflurane^4^^,^^5^ (but not apparently sevoflurane),^6^ a greater surgical demand for relaxation and decrease in functional residual capacity (FRC).^7^^,^^8^ The obese or overweight patient presents kinetic issues that may delay recovery, thereby, aspiration and develop acute upper airway obstruction after tracheal extubation that add to the risks of anesthesia.^9^^-^^11^ Rapid recovery is, therefore, desirable to ensure early efficient coughing and to decrease the rate of postoperative respiratory complications.

One of the elements that determine recovery from anesthesia is the clearance of anesthetic from the effect site. Several factors influence clearance. Anesthetic in tissue depots, the solubility of the anesthetic in blood (the blood/gas partition coefficient) and tissue/blood solubility coefficients^12^^,^^13^ will determine the rate of decrease of anesthetic in the arterial circulation during recovery from anesthesia because solubility determines the clearance of anesthetic at the lungs.^14^ If the fat solubility of the anesthetic is very small, most of the anesthetic will be cleared by ventilation and will thus not recirculate and delay recovery from anesthesia.

Most Iranian sheep have large, fat tails, which accounts for up to 14.5% of the cold carcass weight.^15^ The blood supply to the tail originates from the median sacral artery (MSA) which is a branch of the abdominal aorta.^16^

The objective of the study reported here was to evaluate effectiveness of body fat on recovery times in obese patients. Therefore, the recovery times of anesthesia by isoflurane in intact fat-tailed lambs and lambs with a ligated MSA was conducted.

## Materials and Methods

Seven healthy, 8 to 10-month-old, fat-tailed Lori-Bakhtiyari ewe lambs, with a body condition score of 4 (on a scale of 0 to 5 units)^17^ and with a mean ± SD weight of 34.3 ± 1.2 kg, were used in the present study. The overall health of the sheep was monitored before and throughout the study. The animals were kept in barn and received anti-parasitic medications prior to the study and were acclimatized to the experimental conditions for 14 days. Body weight of the animals was recorded at arrival and before induction of the anesthesia. The animals had free access to hay and tap water throughout the study. Wool was clipped a week before the start of experiment. 

Food was withheld for 20 hr before induction of the anesthesia and the procedure. However the animals had free access to water. All practical parts of the study, including the ligating of MSA, the induction of inhalation anesthesia, and the monitoring of the animals, intra-operative and recovery parameters were performed with the same author. Sheep were anesthetized twice in a two-week interval (the experiment was performed in two stages).

**Stage 1. **Sheep were anesthetized for 4 hr using isoflurane in oxygen. For induction of the anesthesia sheep were restrained gently and isoflurane in 100% oxygen (4 L min^-1^) was delivered via a fitted facemask without using of sedatives or tranquilizers. Prior to anesthesia, the right jugular vein was catheterized for subsequent fluid administration. For induction of the anesthesia, the concentration of isoflurane was increased gradually (0.5% every 30 sec) until a vaporizer setting of 4.5% was reached.

Lidocaine 10% spray (Astra, Sodertalje, Sweden) was used to anesthetize the larynx prior to tracheal intubation. Following intubation, sheep were connected to a re-breathing system and a medium plane of anesthesia, as determined by palpebral and pedal reflexes, was maintained with isoflurane (2.0 to 2.5%) in oxygen (1.5 L min^-1^).

**Stage 2. **Two weeks later, prior to the induction of anesthesia, the MSA of lambs was ligated under epidural analgesia. The level of the lumbosacral junction was prepared aseptically, and the needle placed correctly into the epidural space for injection of 4 to 5 mL of a 2% lidocaine solution (Farvet, Bladel, The Netherlands).

**Fig. 1:**
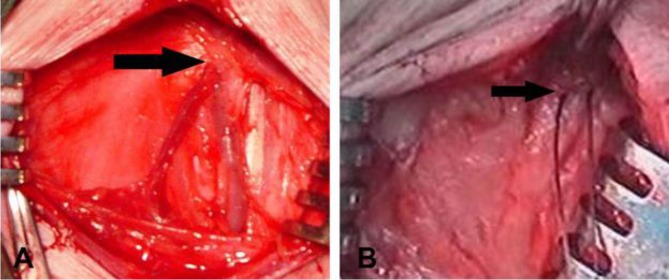
**A) **Identification of the median sacral artery (MSA) after subcutaneous dissection. **B)** Ligation of the MSA with absorbable suture at its most proximal location.

Following epidural anesthesia, sheep were positioned in dorsal recumbency to ligate the MSA. Using aseptic technique, a 4 cm midline incision was made at the base of the tail and after subcutaneous dissection the MSA was identified and ligated using No. 0 chromic catgut at its most proximal location (Figs. 1A and 1B). After careful hemostasis the subcutaneous tissue was closed with No. 0 chromic catgut and the skin with No. 1 polypropylene in a simple continuous pattern. An hour and half after epidural anesthesia, all sheep were anesthetized again as mentioned above. 

Lactated Ringer’s solution (10 mL kg^-1^ per hr) was administered during anesthesia in both stages. 

Heart rate was obtained using a continuous electro-cardiogram monitor (Model RS-2000; Gmedi Co., Seoul, Korea). Rectal temperature was measured at 1 hr intervals. Sheep were placed in left lateral recumbency on a padded surgical table during the maintenance of anesthesia. At the end of anesthesia, sheep were disconnected from the anesthetic circuit and were allowed to breathe room air. Induction time (time from the administration of isoflurane to tracheal intubation), extubation time (time from the discontinuation of isoflurane to swallowing reflex), time to sternal recumbency (time from the discontinuation of isoflurane to sternal recumbency) and attempts to stand (time from the discontinuation of isoflurane to time to standing) were recorded during anesthetic induction and recovery.

**Data analysis.** Experimental results were expressed as mean ± standard deviation (SD). All data were analyzed by one-way analysis of variance to assess statistical significance between experimental groups using SPSS (Version 17; SPSS Inc., Chicago, USA) and *p* value less than 0.05 was considered significant.

## Results

It was relatively easy to locate the MSA at the base of the tail. In the stage 2, ligation of the MSA was performed without difficulties and no complications were encountered during surgery or post operation.

Mask induction was easily performed in sheep with minimal restraint. The induction of anesthesia was smooth and no struggling or objection to placement of the mask was observed.

**Fig. 2:**
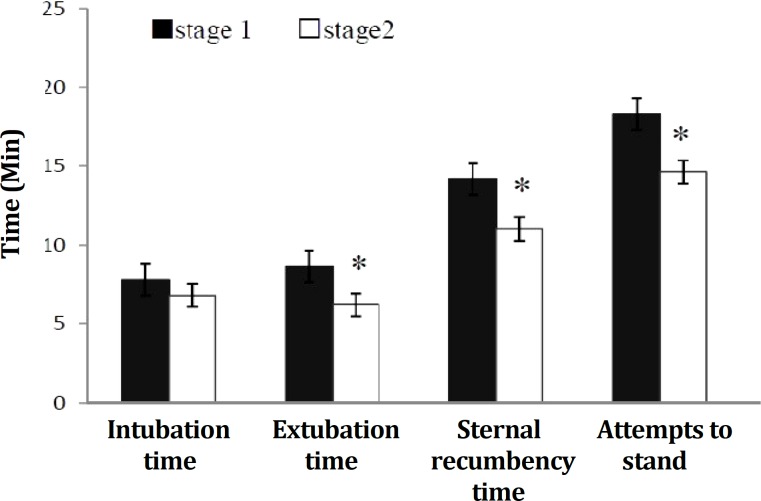
Induction and recovery times in stage 1 (n=7) and stage 2, median sacral artery (MSA)-ligated sheep (n=7) following 4 hr isoflurane anesthesia.

Times to induce, extubate, sternal recumbency and attempts to stand are shown in Fig. 2. No significant differences were observed for the induction time between groups (*p* > 0.05). However, extubation, sternal recumbency and attempts to stand times were significantly longer in intact sheep (*p* < 0.05).

In both stages significant decreases in rectal temperature were observed in both groups, no significant changes in heart rate or rhythm were detected (Table 1).

Increased salivation was occurred in most sheep during isoflurane anesthesia. One sheep in the control group showed moderate bloat during the first stage of the experiment. All sheep were recovered uneventfully from anesthesia and no adverse reactions or complications were encountered in the present study.

**Table 1:** Mean (SD) cardiopulmonary measurements and body temperatures of seven sheep anesthetized with isoflurane at intervals after the induction of anesthesia in two stages

**Groups**	**Parameters**	**Time from induction of anesthesia (Min)**			
		**60**	**120**	**180**	**240**
Stage 1	HR (bpm)	82.0 ± 15.5	78.5 ± 13.5	77.0 ± 14.5	75.2 ± 13.7
Stage 1	TEMP (˚C)	38.6 ± 0.5	38.0 ± 0.7	37.4 ± 0.7	37.0 ± 0.7
Stage 2	HR (bpm)	83.0 ± 16	80.3 ± 13.2	78.0 ± 15.1	76.0 ± 13.5
Stage 2	TEMP (˚C)	38.7 ± 0.5	38.1 ± 0.6	37.3 ± 0.7	37.1 ± 0.6

HR: Heart rate, TEMP: Temperature.

There were no significant differences between these data (*p *> 0.05).

* indicate significant differences at *p* < 0.05 compared to stage 1.

## Discussion

The results of this study supported the hypothesis that body fat is effective on recovery times in obese patients after prolonged periods of anesthesia. In the present study, anesthesia was induced with isoflurane delivered by mask, so any possible effects of premedications or injectable anesthetic agents on recovery times could be eliminated. Theoretical considerations suggest that if the inhalation anesthetics had been used without other medication, recovery should have been faster with desflurane, followed by sevoflurane and slower with isoflurane, but in experimental study Mohammadnia *et al*. compared the three inhalation agents in sheep after injection of xylazine as premedication agent that there were no differences between desflurane, sevoflurane and isoflurane in time of recovery.^19^ Although we did not measure the inhaled concentration of isoflurane, the vaporizer setting and the depth of anesthesia was the same in both groups. Because no surgery was performed during the anesthesia, it was easy to maintain a constant light level of anesthesia throughout the 4 hr period of anesthesia.

Fat bears a large capacity for anesthetic storage and may serve as a reservoir in obese patients. The impact of anesthetic stored in fat may be the result of a return of the anesthetic in blood perfusing the fat or of a transfer from fat to adjacent highly perfused tissues (e.g., omental/ mesenteric fat to intestine and liver).^14^ Such accumulation may delay recovery from anesthesia.

On average, the tail weight is 12.6% of carcass weight in Lori-Bakhtiyari rams.^15^ Therefore, the role of approximately 4.25 kg fat in anesthetic storage was abated by MSA-ligation. During the two weeks (Stage 1 to 2), on average, one kg was added to bodyweight of each sheep.

In this study the indices used to assess recovery were extubation, sternal recumbency and attempts to stand. For all species these include extubation,^18^ however, in human being they include awareness factors.^1^ In most veterinary studies final recovery is taken as the ability of the animal to stand, unless, standing might be limited such as orthopedic reasons.^18^^,^^19^ Final recovery in the present study was taken as attempts to stand .When recovering from anesthesia, sheep should not be left unattended until they can safely maintain themselves in sternal recumbency; preliminary pilot studies showed that sheep could do attempts to stand, once they were sufficiently awake.

Time to extubate, sternal recumbency and attempts to stand were significantly shorter in MSA-ligated sheep. Also, in our previous experimental study, we showed that the peak serum fluoride concentration and recovery time from anesthesia with halothane were decreased following ligation of MSA in fat-tailed sheep.^20^ Shorter recovery time has been reported in sheep without a fat tail.^21^ The more rapid recovery times may be attributed to the faster decline in the alveolar partial pressure of isoflurane in MSA-ligated sheep. We hypothesize that the rate of alveolar washout is faster in MSA-ligated sheep. Increased recovery time has also been observed in obese human patients compared with non-obese patients.^22^ Also Juvin *et al*.,^23^ showed that recovery was more rapid in such patients when they were anesthetized with desflurane versus isoflurane.

The blood-gas partition coefficient represents the partial pressure or solubility of an anesthetic between blood and gas at equilibrium. The higher the value of blood-gas partition coefficient, the greater the solubility of an anesthetic in the blood and vice versa and the lower the value, the lower the blood solubility of the anesthetic.^19^

Isoflurane is available as a clear nonflammable, halogenated methyl ethyl ether with a blood/gas partition coefficient of 1.4 and fat/blood partition coefficient of 45.0. Its intermediate blood solubility rating along with its relatively high lipophilicity indicates that this agent will produce a fairly rapid induction.^24^

Intermediate fat solubility of isoflurane (fat/gas partition coefficient approximately 45.0) limits elimination into alveolar gas. This implies that the total amount of isoflurane extracted by fat tissue is higher in intact sheep compared with MSA-ligated sheep.

Studies showed that the newest volatile anesthetics such desflurane and sevoflurane, have significantly lower blood/gas partition coefficients (0.4 and 0.6, desflurane versus sevoflurane, respectively) than isoflurane (1.4) or halothane (2.4), predicting better intraoperative control of anesthesia and a more rapid recovery from anesthesia.^12^^,^^13^^,^^25^^,^^26^

 In conclusion, recovery times decreased following MSA ligation in fat-tailed sheep, which suggested that body fat had a major role in recovery times of isoflurane in sheep after prolonged periods of anesthesia. We developed an animal model to investigate fat drug solubility of isoflurane gas. Therefore, using of less-soluble in fat anesthetics is better than high-soluble anesthetics for prolonged anesthesia to decrease postoperative complication.

## References

[1] Eichenberger A, Proietti S, Wicky S (2002). Morbid obesity and postoperative pulmonary atelectasis: an underestimated problem. Anesth Analg.

[2] Hood EE, Dewan DM (1993). Anesthetic and obstetric outcome in morbidly obese parturients. Anesthesiology.

[3] Auler JO Jr, Miyoshi E, Fernandes CR (2002). The effects of abdominal opening on respiratory mechanics during general anesthesia in normal and morbidly obese patients: A comparative study. Anesth Analg.

[4] Bentley JB, Vaughan RW, Gandolfi AJ (1982). Halothane biotransformation in obese and non-obese patients. Anesthesiology.

[5] Bentley JB, Vaughan R, Miller MS (1979). Serum inorganic fluoride levels in obese patients during and after enflurane anesthesia. Anesth Analg.

[6] Frink EJ, Malan TP, Brown E (1993). Plasma inorganic fluoride levels with sevoflurane anesthesia in morbidly obese and non-obese patients. Anesth Analg.

[7] Damia G, Mascheroni D, Croci M (1988). Perioperative changes in functional residual capacity in morbidly obese patients. Br J Anesth.

[8] Ogunnaike BO, Jones SB, Jones DB (2002). Anesthetic considerations for bariatric surgery. Anesth Analg.

[9] Rose DK, Cohen MM, Wigglesworth DF (1994). Critical respiratory events in the postanesthesia care unit. Anesthesiology.

[10] Schwartz AR, Patil SP, Laffan AM (2008). Obesity and obstructive sleep apnea: pathogenic mechanisms and therapeutic approaches. Proc Am Thorac Soc.

[11] Torri G, Casati A, Albertin A (2001). Randomized comparison of isoflurane and sevoflurane for laparoscopic gastric banding in morbidly obese patients. J Clin Anesth.

[12] Malviya S, Lerman J (1990). The blood/gas solubilities of sevoflurane, isoflurane, halothane, and serum constituent concentrations in neonates and adults. Anesthesiology.

[13] Yasuda N, Targ AG, Eger EI II (1989). Solubility of I-653, sevoflurane, isoflurane, and halothane in human tissues. Anesth Analg.

[14] Eger E I, Saidman L J (2005). Illustrations of inhaled anesthetic uptake, including intertissue diffusion to and from fat. Anesth Analg.

[15] Kiyanzad M R (2005). Comparison of carcass composition of Iranian fat-tailed sheep. Asian-Aust J Anim Sci.

[16] Chahrasbi H, Radhmer B (1975). Anatomical peculiarities of body fat (Adipose) tail of the sheep breeds in Iran [French]. Chah de Med Vet.

[17] Rankins D L, Ruffin DC, Pugh DG, Pugh DG (2002). Feeding and nutrition. Sheep and goat medicine.

[18] Mohamadnia AR, Hughes G, Clarke KW (2008). Maintenance of anesthesia in sheep with isoflurane, desflurane or sevoflurane. Vet Rec.

[19] Sellers G, Lin HC, Chamorro MF (2013). Comparison of isoflurane and sevoflurane anesthesia in Holstein calves for placement of portal and jugular vein cannulas. Am J Anim Vet Sci.

[20] Sharifi S, Vesal N (2005). Effects of tail fat on halothane biotransformation in fat-tailed sheep. Clin Exp Pharmacol P.

[21] Dehghani SN, Varshowi HR (1995). Effects of tail fat on induction and recovery of sheep anesthetized with halothane. Small Ruminant Res.

[22] Saraiva RA, Lunn JN, Mapleson WW (1977). Adiposity and pharmacokinetics of halothane The effect of adiposity on the maintenance of and recovery from halothane anesthesia. Anesthesia.

[23] Juvin P, Vadam C, Malek L (2000). Postoperative recovery after desflurane, propofol, or isoflurane anesthesia among morbidly obese patients: a prospective, randomized study. Anesth Analg.

[24] SteffeyEP, Mama KR, Tranquilli WJ, Thurmon JC, Grimm KA (2007). Inhalation Anesthetics. Veterinary anesthesia and analgesia.

[25] Eger EI (1987). Partition coefficients of I-653 in human blood, saline, and olive oil. Anesth Analg.

[26] Strum DP, Eger EI (1987). Partition coefficients for sevoflurane in human blood, saline, and olive oil. Anesth Analg.

